# Narciclasine, a novel topoisomerase I inhibitor, exhibited potent anti-cancer activity against cancer cells

**DOI:** 10.1007/s13659-023-00392-1

**Published:** 2023-08-29

**Authors:** Meichen Wang, Leilei Liang, Rong Wang, Shutao Jia, Chang Xu, Yuting Wang, Min Luo, Qiqi Lin, Min Yang, Hongyu Zhou, Dandan Liu, Chen Qing

**Affiliations:** 1 School of Pharmaceutical Science and Yunnan Key Laboratory of Pharmacology for Natural Products, Kunming Medical University, 1168 Western Chunrong Road, Yuhua Street, Cheng Gong District, Kunming, 650500 Yunnan People’s Republic of China; 2 Cell Biology and Molecular Biology Laboratory of Experimental Teaching Center, Faculty of Basic Medical Science, Kunming Medical University, 1168 Western Chunrong Road, Yuhua Street, Cheng Gong District, Kunming, 650500 Yunnan China; 3 Yunnan Infectious Disease Hospital, 28 km at Shi’an Road, Taiping Town, Anning, Kunming, 650301 Yunnan China

**Keywords:** Topoisomerase, Narciclasine (NCS), Topo I-DNA covalent complex, DNA damage, Cell cycle, Apoptosis

## Abstract

DNA topoisomerases are essential nuclear enzymes in correcting topological DNA errors and maintaining DNA integrity. Topoisomerase inhibitors are a significant class of cancer chemotherapeutics with a definite curative effect. Natural products are a rich source of lead compounds for drug discovery, including anti-tumor drugs. In this study, we found that narciclasine (NCS), an amaryllidaceae alkaloid, is a novel inhibitor of topoisomerase I (topo I). Our data demonstrated that NCS inhibited topo I activity and reversed its unwinding effect on p-HOT DNA substrate. However, it had no obvious effect on topo II activity. The molecular mechanism of NCS inhibited topo I showed that NCS did not stabilize topo-DNA covalent complexes in cells, indicating that NCS is not a topo I poison. A blind docking result showed that NCS could bind to topo I, suggesting that NCS might be a topo I suppressor. Additionally, NCS exhibited a potent anti-proliferation effect in various cancer cells. NCS arrested the cell cycle at G_2_/M phase and induced cell apoptosis. Our study reveals the antitumor mechanisms of NCS and provides a good foundation for the development of anti-cancer drugs based on topo I inhibition.

**Graphical abstract:**

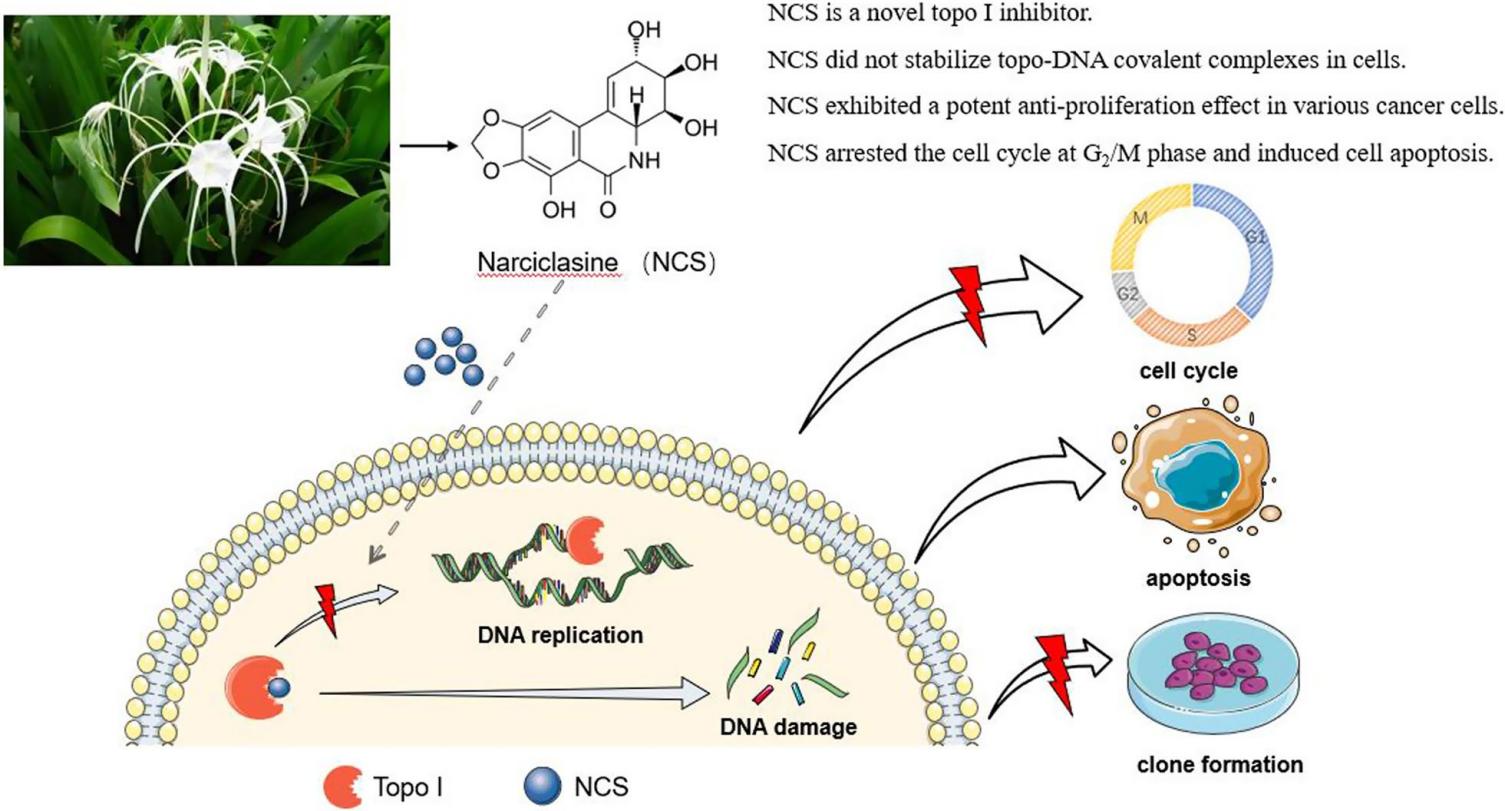

## Introduction

Malignant tumors have become a major chronic disease that seriously endangers the lives and health of people worldwide. According to the data from International Agency for Research on Cancer (IARC), the global cancer burden will increase to 19.3 million new cases and 10 million deaths in 2020 [1]. Natural products are a rich source of lead compounds for drug discovery, including anti-tumor drugs [2]. Currently, several clinically useful anti-cancer agents were derived from plants, including camptothecin (CPT) derivatives-topotecan and irinotecan, paclitaxel and vinblastine [3]. CPT and its derivatives, which specifically targets topoisomerase I (topo I), are one of the most popular plant-derived anticancer agents of clinical significance [4].

Topoisomerase inhibitors are a series of chemotherapeutics with a definite curative effect, and their mechanism of action is to inhibit the activity of topoisomerase in tumor cells. Briefly, the presence of topoisomerase releases excess tension and maintains DNA integrity [5]. Topoisomerase inhibitors inhibit the activity of topoisomerase, so that the base tension during the DNA replication process cannot be released and eventually leads to cell death. Topoisomerase inhibitors can be divided into two categories according to the different mechanism of action: topoisomerase suppressor and topoisomerase poison. Topoisomerase suppressor leads to the death of tumor cells by inhibiting topoisomerase activity, while topoisomerase poison combines with the topoisomerase I-DNA cleavage complex (topo I-DNA CC) to form a ternary complex to stabilize the cleavage complex. There is an irreversible DNA double-strand break damage (DSB) when the ternary complex collides with the DNA replication fork, which eventually leads to cell death. In the 1870s, CPT was used as an anti-tumor compound for clinical research for the first time. Fifteen years later, it was discovered that the target of CPT was topo I [6]. In 1996, the Food and Drug Administration (FDA) approved two CPT derivatives, irinotecan and topotecan, as topo I inhibitors. Taking irinotecan as an example, it can be used for the clinical treatment of various cancer, such as lung cancer [7–10], colorectal cancer [11], ovarian cancer [12, 13], gastric cancer [14] and cervical cancer [15]. And the sensitivity of cancer cells to irinotecan mainly depends on the expression of topoisomerase in the cells [15]. In recent years, a tumor-targeted drug delivery strategy has achieved outstanding results in clinical development, including liposome or nanoparticle preparations (MM-398, onivyde®) [16] and monoclonal antibody-conjugated preparations (ADCs) (IMMU-132, DS-8201) [17, 18].

**Fig. 1:**
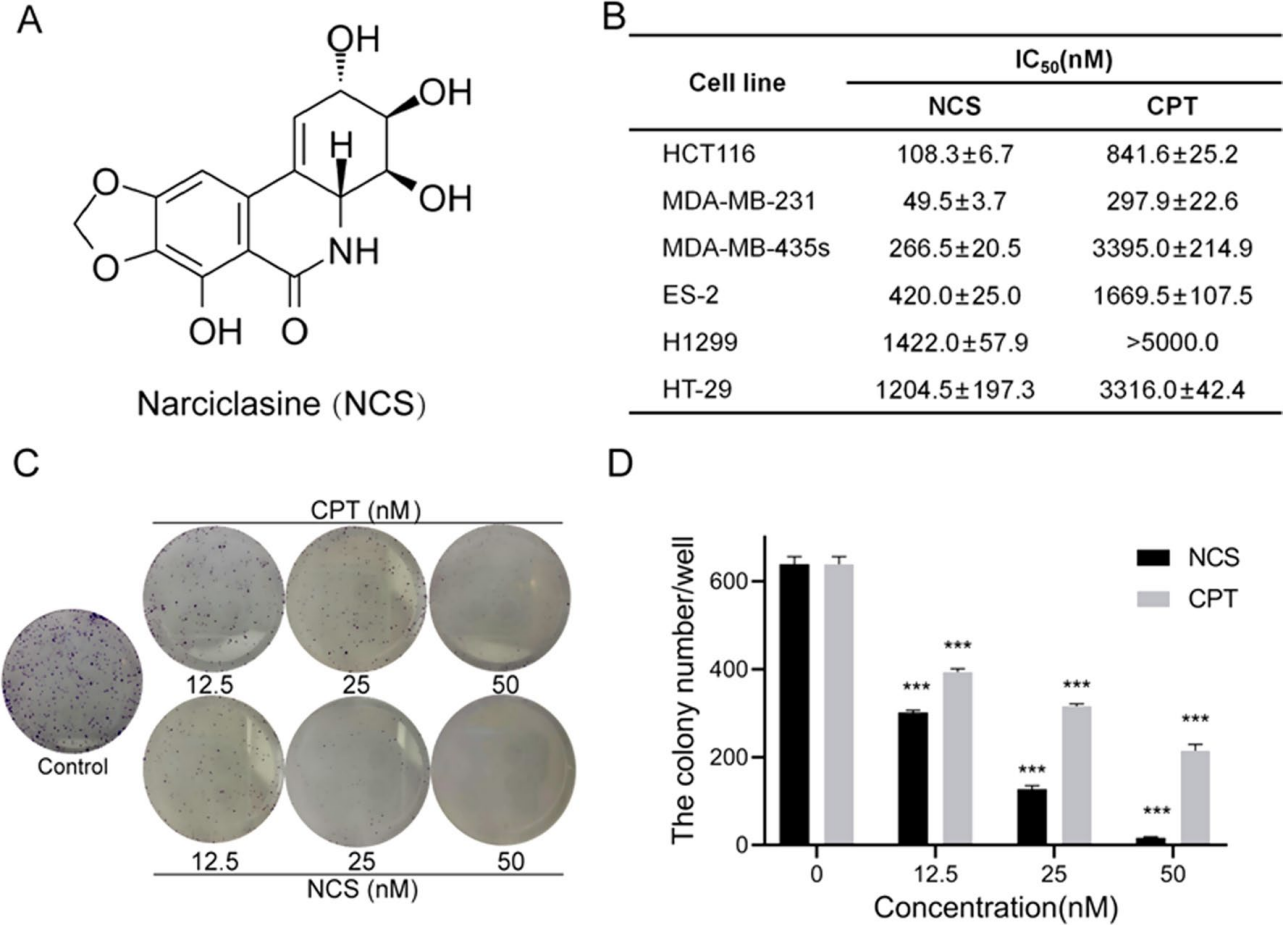
NCS could effectively decrease cell viability and inhibit cell proliferation in different tumor cells. **A** The chemical structure of NCS. **B** The IC_50_ values of NCS and CPT in different cancer cell lines. **C** Clone formation assay was performed in MDA-MB-231 cells. The cells were respectively treated with different concentrations of CPT and NCS for ten days, and were stained by 0.1% crystal violet. The images were acquired under the microscope. **D** Quantitative analysis of the colony number in each well was performed. Data were represented as the means ± SD (n = 3). ***P < 0.001 difference versus vehicle-treated control cells

The plant family Amaryllidaceae is distinguished for its alkaloid principles which exhibit selective cytotoxicity against cancer cells [19]. Narciclasine (NCS, Fig. 1A), a member of amaryllidaceae alkaloids, has been proven to exhibit potent antitumor effect against different human cancer cells [19, 20]. One study on six kinds of amaryllidaceae alkaloids reported that NCS exhibited the most significant inhibitory effect on the growth of tumor cells [21]. Another study of NCS on sixty human cells showed that NCS had a strong and selective cytotoxic effect on tumor cells in the low concentration range [22]. Mechanism studies showed that NCS could interact with the ribosomal 60s subunit and inhibit the formation of peptide bonds by preventing the 3’ end of the donor substrate from binding to the peptidyl transferase center, thereby reducing protein synthesis [23, 24]. Another study reported that NCS induced apoptosis by affecting the AMPK-ULK1 signaling pathway in triple-negative breast cancer cell lines [25]. In recent years, studies have been performed to investigate the antitumor potency and mechanism of NCS and suggested that NCS might be a promising candidate for the development of antitumor drugs. However, the underlying mechanisms and the antitumor targets of NCS remains largely unknown.

In this study, we found that NCS is a novel topo I inhibitor. NCS inhibited topo I activity and reversed its unwinding effect on p-HOT DNA substrate, but had no obvious effect on topo II activity. The molecular mechanism by which NCS inhibited topo I showed that NCS did not stabilize topo-DNA covalent complexes in cells, indicating that NCS is not a topo I poison. A blind docking result showed that NCS could bind to topo I, suggesting that NCS might be a topo I suppressor. Additionally, NCS arrested the cell cycle at G_2_/M phase and induced cell apoptosis. Taken together, our present study revealed the antitumor mechanisms of NCS and laid a good foundation for the development of anti-cancer drugs based on topo I inhibition.

## Result and discussion

### NCS decreased the cell viability of different cancer cells

The anti-proliferative potency of NCS and CPT was examined in a panel of cells derived from different cancer types by MTT assay and clone formation assay. The sensitivity of different human cancer cells to NCS and CPT was compared by half-maximal inhibitory concentration (IC_50_) values (Fig. 1B), showing that NCS exhibited a greater inhibitory effect than CPT in various cancer cells. Among all the cancer cell lines, NCS had a greater inhibition on MDA-MB-231 cells than others (Fig. 1B). Therefore, MDA-MB-231 cells were selected for further experiments in our study. Results of clone formation assay showed that NCS could inhibit the clonal formation of cancer cells in a concentration-dependent manner (Fig. 1C, D). The above data indicated that NCS had anti-cancer activity against different cancer cells.

### NCS induced G_2_/M phase cell cycle arrest

**Fig. 2:**
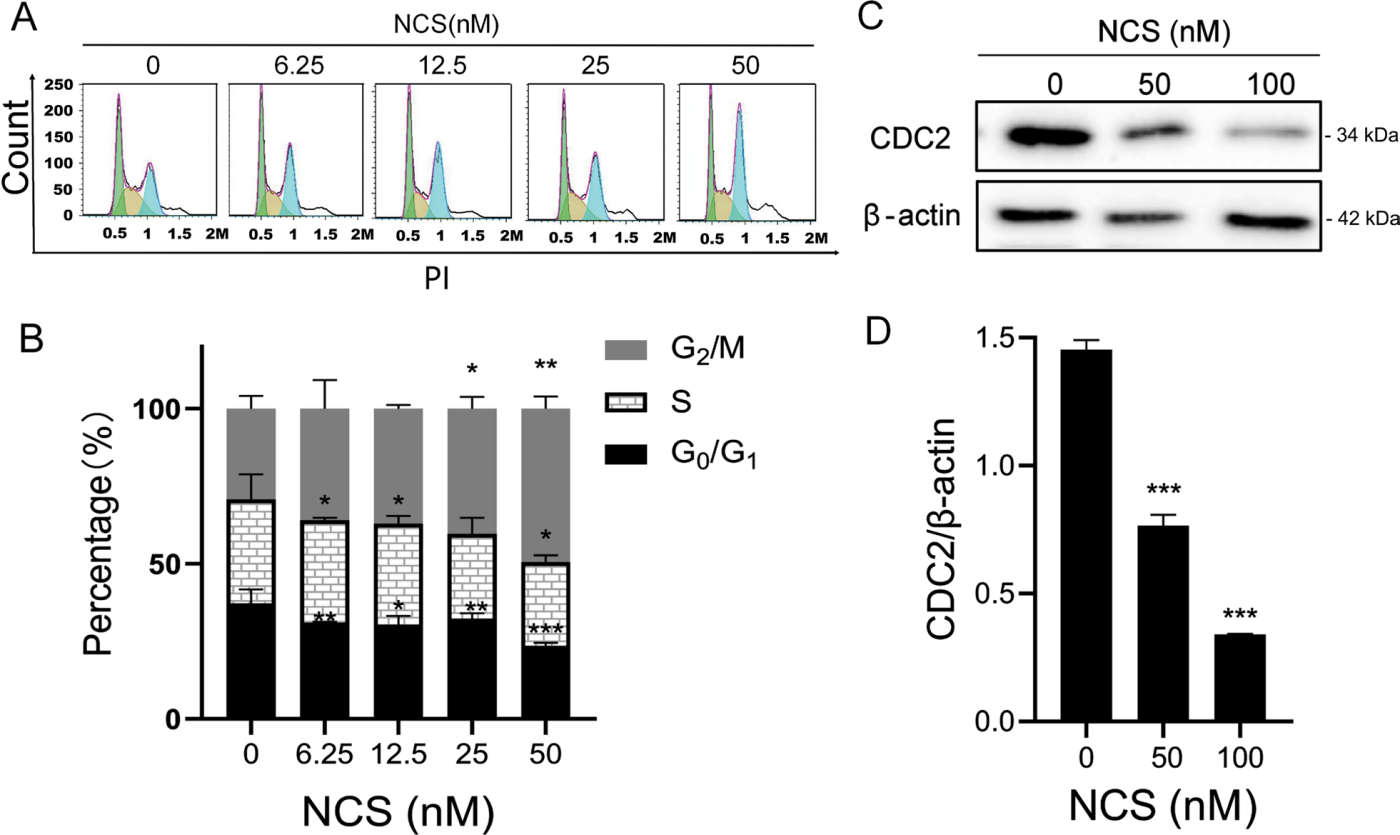
NCS induced cell-cycle arrest in G_2_/M phase. **A** MDA-MB-231 cells were treated with NCS at indicated concentrations for 24 h, and then subjected to cell cycle analysis. **B** Quantitative analysis of cells in each cell cycle phase was performed. Results are presented as mean ± SD (n = 3). *P < 0.05, **P < 0.01, ***P < 0.001 difference versus vehicle-treated control group. **C** The inhibition of CDC2 expression contributed to NCS-induced G_2_/M phase arrest. MDA-MB-231 cells were treated with indicated concentrations of NCS for 4 h, the expression of CDC2 was analyzed by western blotting analysis. β-actin was used as a loading control. **D** Blots for indicated protein expressions were semi-quantified using ImageJ software. Data were represented as the means ± SD (n = 3). ***P < 0.001 difference versus vehicle-treated control cells

To investigate how NCS inhibited cell proliferation, we assessed the effect of NCS on cell cycle distribution by using propidium iodide (PI) staining and flow cytometry. As shown in Fig. 2A, treatment of NCS in MDA-MB-231 cells induced G_2_/M phase cell accumulation in a concentration-dependent manner. The proportion of the cell population in G_2_/M phase increased from 25.4 ± 3.5% to 38.3 ± 3.07% after NCS treatment (Fig. 2B). CDC2, known as cyclin-dependent kinases 1 (CDK1), is a key regulator for G_2_ to M phase transition. To further understand the mechanism by which NCS induced cell cycle arrest, the protein level of CDC2 was determined by western blot analysis. The results showed that treatment of the cells with NCS for 4 h decreased the expression of CDC2 in a concentration-dependent manner (Fig. 2C, D). Therefore, the above results demonstrated that NCS arrested cells in G_2_/M phase of cell cycle, with the involvement of decreasing CDC2 expression.

### NCS induced tumor cell apoptosis

**Fig. 3:**
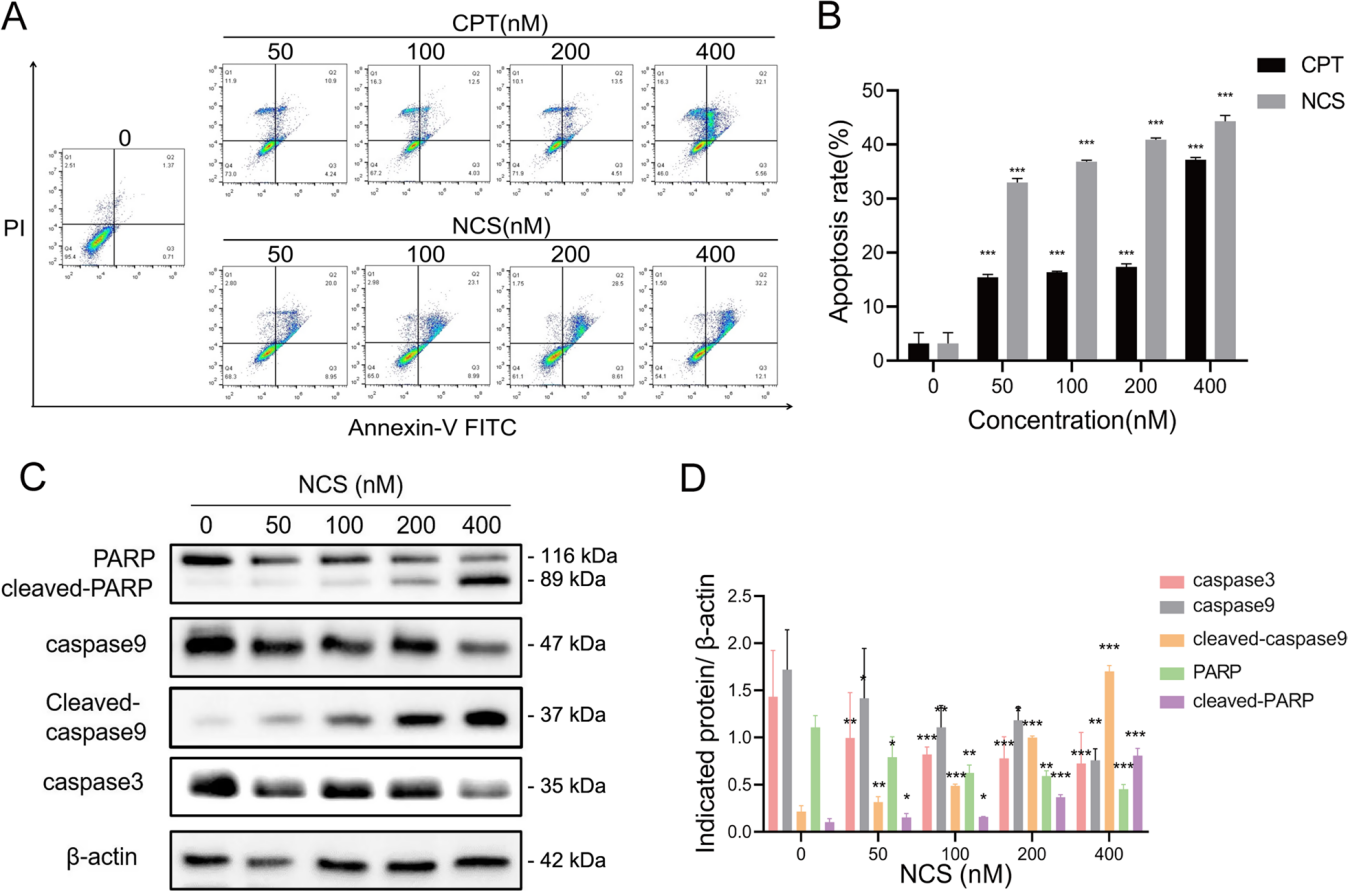
NCS induced apoptosis in MDA-MB-231 cells. **A** MDA-MB-231 cells were treated with indicated concentrations of NCS for 48 h, then was subjected to Annexin V-FITC/PI staining and flow cytometry analysis to determine cell apoptosis rate. **B** Quantitative analysis of cell apoptosis rate was performed. Results are presented as mean ± SD (n = 3). ***P < 0.001 difference versus vehicle-treated control group. **C** MDA-MB-231 cells were treated with indicated concentrations of NCS for 4 h. The indicated proteins were detected by western blot analysis. **D** Blots for indicated protein expressions were semi-quantified using ImageJ software. Data were represented as the means ± SD (n = 3). *P < 0.05, **P < 0.01, ***P < 0.001 difference versus control cells

To investigate the pro-apoptotic activity of NCS in cancer cells, apoptosis rates were measured by flow cytometry. As shown in Fig. 3A, a strong pro-apoptotic effect was observed in NCS-treated cells after 48 h of treatment. In MDA-MB-231 cells, apoptosis rates significantly increased from 3.17 ± 2.01% to 44.3 ± 1.1% after the treatment with different concentrations of NCS (Fig. 3B). Caspase-9 (initiator caspases) and caspase-3 (executioner caspases) are upstream and downstream of the caspase cascade in the endogenous apoptotic pathway, respectively [26]. PARP is a substrate of activated caspase-3. Cleaved caspase-3 cuts PARP to inactivate it which accelerate cells instability. Thus successful cleavage of PARP is a hallmark of caspase-3 activation [27–29]. Western blot analysis showed that PARP and caspase-9 cleavages were enhanced, and caspase-3, 9 and PARP prototypes were decreased after NCS treatment (Fig. 3C, D), indicating that NCS initiated apoptosis in tumor cells by activating the caspase cascade.

### NCS induced DNA damage in tumor cells but did not directly interact with DNA

**Fig. 4:**
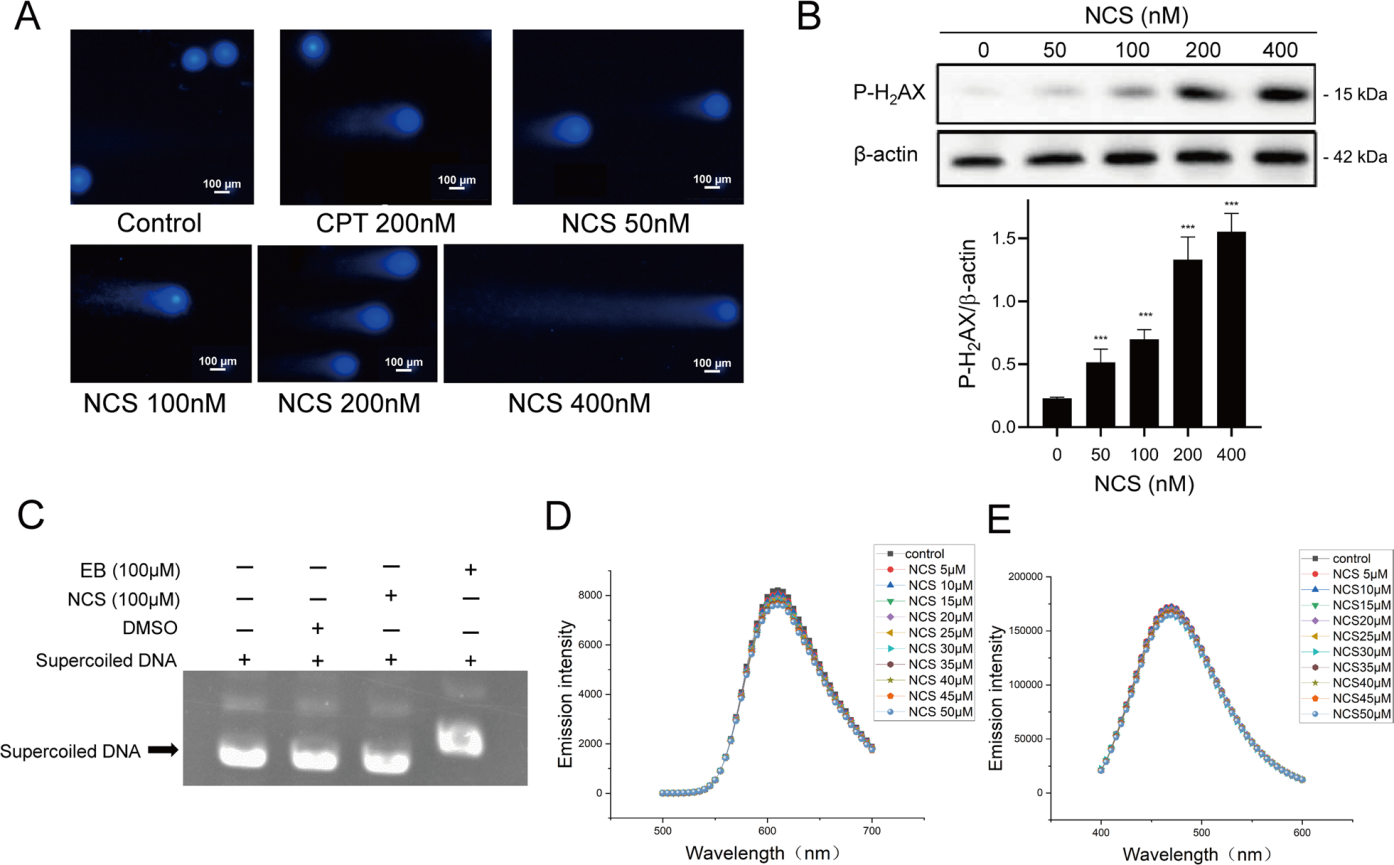
NCS induced DNA damage in MDA-MB-231 cells. **A** MDA-MB-231 cells were treated with indicated concentrations of NCS for 4 h, then was subjected to commet assay. **B** MDA-MB-231 cells were treated with indicated concentrations of NCS for 4 h. The indicated proteins were detected by western blot analysis. Blots for indicated protein expressions were semi-quantified using ImageJ software. Data were represented as the means ± SD (n = 3). ***P < 0.001 difference versus control cells. **C** DNA cleavage assay was to detect whether NCS could cause p-HOT DNA breakage directly. **D** EB fluorescence competition assay, and ctDNA worked as the substrate. (ctDNA/ EB = 4) **E** Hoechst 33258 fluorescence competition assay, and ctDNA worked as the substrate. (ctDNA/Hoechst = 32).

DNA damage is a commonly involved in cell cycle arrest and inhibition of cell proliferation. To further investigate the underlying mechanism by which NCS exhibited anti-tumor activity, the effect of NCS on cellular DNA was determined by comet tailing assay. As shown in Fig. 4A, with the concentration of NCS increased, the longer and more obvious comet tailing was detected in NCS-treated tumor cells, indicating that NCS induced DNA damage in a concentration-dependent manner. Elevated of H_2_AX phosphorylation is widely used as a hallmark of DNA damage [30]. By western blot analysis, we found that NCS significantly increased the phosphorylation of H_2_AX (Fig. 4B), a marker of DNA damage, further demonstrating that NCS could induce DNA damage.

To distinguish whether NCS caused DNA damage by interacting with DNA substrate or through other indirect way, we firstly performed extracellular DNA cleavage assay by directly incubating p-HOT with NCS. Electrophoresis experiment data showed that a single band corresponding to supercoiled DNA was observed in the 100 µM NCS group, suggesting that NCS did not exhibit evident cleavage ability (Fig. 4C). In contrast, EB which can effectively insert into DNA base pairs reduced the substrate DNA mobility, while NCS could not. The above data suggested that NCS might not directly interact with DNA.

Ethidium bromide (EB) is a fluorescent probe that exhibits drastically enhanced fluorescence intensity upon intercalation within DNA [31]. If a compound is added and results in DNA-induced quenching of EB fluorescence emission, it indicates that the compound can replace EB and insert into DNA. Hoechst 33258, as a DNA minor groove binder, can be used for this test. Therefore, we used EB/Hoechst 33258-DNA fluorescence competition assays to determine whether NCS can replace EB or Hoechst 33258 to bind to DNA. With continuous addition of NCS to the EB/Hoechst 33258-DNA system, the emission fluorescence spectrums did not change significantly (Fig. 4D, E), indicating that NCS was neither a DNA intercalator nor a DNA groove binder. Taken together, the above results suggest that NCS did not directly interact with DNA, and NCS-induced DNA damage may occur through other indirect mechanisms (such as topo I inhibition).

### NCS inhibited Topo I enzyme activity

**Fig. 5:**
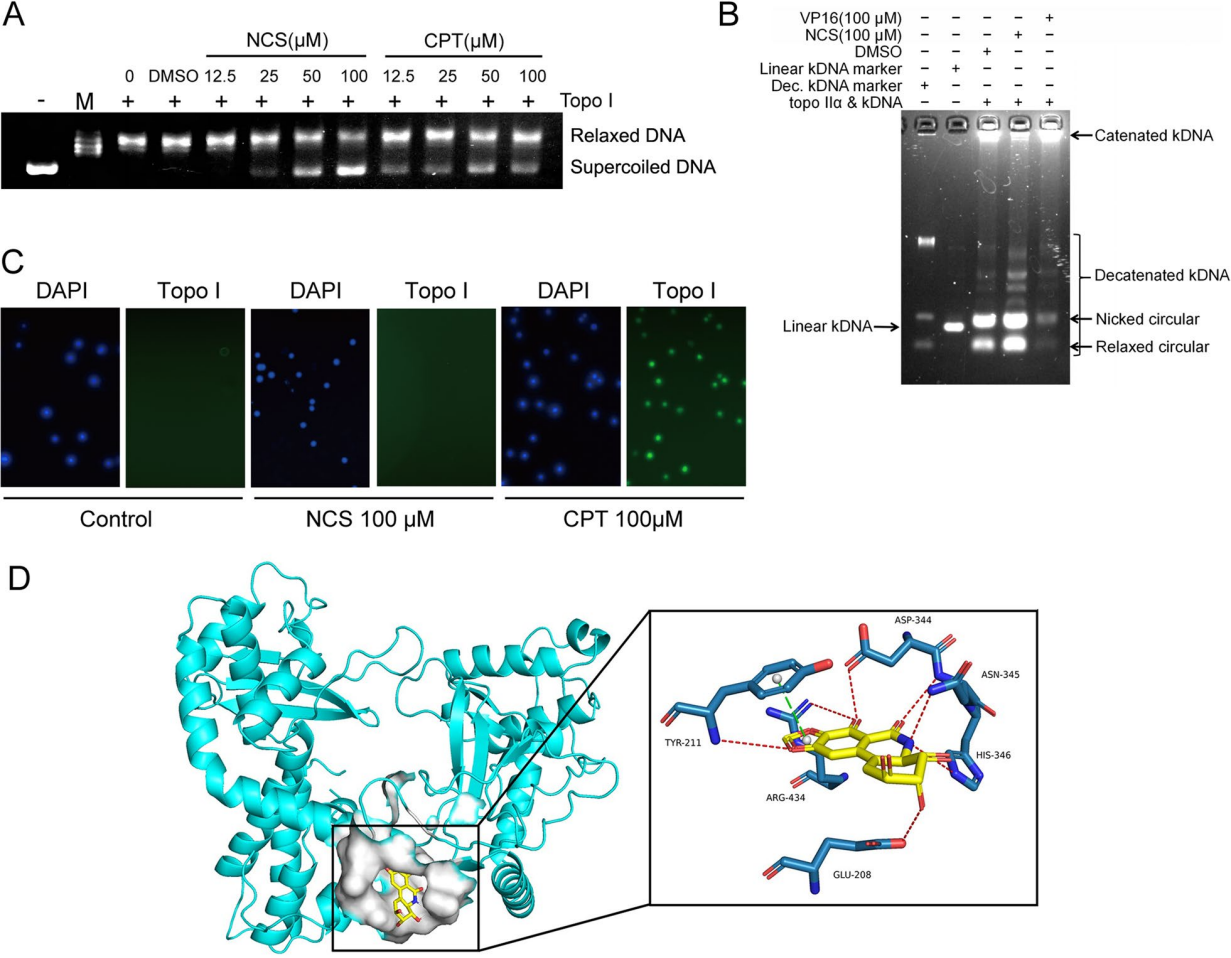
NCS is not a topo I poison but a topo I suppressor. **A**, **B** The effects of NCS on topo I **A** and topo II **B** activities were examined by topo I mediated DNA relaxation assay and topo II mediated kDNA decatenation assay using TopoGEN’s topo I and topo II assay kits. **C** TARDIS assay was performed to quantify topo I-DNA complexes that resulted from the actions of topoisomerase poison. The green fluorescent spot formed by the ternary complex and the blue spot formed by the DNA in the nucleus. **D** 3D representation of the predicted binding mode and intermolecular interactions between topo I (PDB ID: 1EJ9) and NCS. Red dash represents hydrogen bonding interaction, green dash represents π-π stacking

DNA topoisomerases are highly specialized nuclear enzymes involved in the correction of topological DNA errors and maintaining DNA integrity, have been proved to be promising targets of antitumor drugs. In our study, topo I mediated DNA relaxation assay and topo II mediated kDNA decatenation assay in cell-free system were applied to determine the inhibitory effect of NCS on topo I and topo II [32, 33]. As shown in Fig. 5A, supercoiled DNA was relaxed by topo I in the absence of NCS. In contrast, the supercoiled DNA accumulated and uncoiled DNA decreased when topo I-DNA system were treated with increasing concentrations of NCS, indicating that NCS inhibited the relaxation effect of topo I on DNA (Fig. 5A). The positive control, CPT, also inhibited topo I activity, but its effect was weaker than that of NCS. In topo IIα decatenation experiments, catenated kDNA was decatenated to nicked circular kDNA and relaxed circular kDNA under the action of topo IIα [34]. NCS (100 µM) was incubated with kDNA in the presence of topo IIα, and kDNA was still efficiently decatenated, indicating that NCS did not effectively inhibit topo IIα (Fig. 5B). The above results clearly demonstrated that NCS is a topo I inhibitor.

### NCS is not a topoisomerase poison

Topo I inhibitors are broadly divided into topo I suppressor and topo I poison. Topo I poison works by stabilizing topo I-DNA complexes. To determine whether NCS is a topo I suppressor or a topo I poison, the trapped in agarose immunostaining (TARDIS) assay [35] was used to quantify topo I-DNA complexes resulting from the actions of topo I poison. Cells treated with NCS or CPT were immobilized in an agarose gel, and the gel was submerged in lysis buffers. The genomic DNA and topo I covalently linked to the DNA remained in the agarose. Topo I and DNA were stained by immunofluorescence and 4’,6-diamidino-2-phenylindole (DAPI), respectively. Location and intensity of fluorescence signals showed the ability of compounds to stabilize topo I-DNA complexes. In the TARDIS assay, the results showed that CPT, a topoisomerase poison, captured the green fluorescent spot formed by topo I in the ternary complex and the blue spot formed by the DNA in the nucleus (Fig. 5C). However, NCS did not captured green fluorescent spot, showing similar effect with the control group (Fig. 5C). The above results proved that NCS could not stabilize topo I-DNA complexes, so it was not a topological poison and might be a topo I suppressor.

To further predict how NCS functioned as a topo I suppressor, a blind docking was performed by Qvina-W and the interactions between NCS and topo I was analyzed and visualized using pymol [36, 37]. The binding pose with the best docking score (-7.7 kcal/mol) was shown in Fig. 5D, and the result indicated NCS tends to bind with topo I in a pocket composed of residues TYR211, ARG434, GLU208, ASP344, ASN345 and HIS346. NCS can form a lot of hydrogen bonding interactions with GLU208 (2.35 Å), TYR211 (3.73 Å), ASP344 (2.02 Å), ASN345 (2.09 Å, 2.26 Å), HIS346 (3.05 Å) and ARG434 (2.96 Å, 2.76 Å), along with π-π stacking interactions with TYR211 (3.73 Å) (Fig. 5D). The above result further suggests that NCS might be a topo I suppressor.

## Experimental section

### Chemicals and reagents

Antibodies against CDC2 (#9116), PARP (#9532), caspase9 (#9504), cleaved-caspase9 (#9509), caspase3 (#9662), p-H_2_AX (#80,312) and β-actin (#3700) were purchased from Cell Signaling Technology (USA). Antibodies against topo I (sc-6903) were purchased from Novus Biologicals (USA). Horseradish peroxidase–conjugated secondary antibodies against mouse (sc-2748) and rabbit (sc-2750) were purchased from Santa Cruz and Alexa Fluor® 488-conjugated secondary antibodies against Rabbit IgG (151,170) were purchased from Jackson ImmunoResearch (USA).

NCS and CPT was purchased from Tauto Biotech (Shanghai, China) and Adamas Reagent Co., Ltd. (Shanghai, China), respectively. Protease inhibitor, phosphatase inhibitor, ct-DNA, and MTT were purchased from Sigma-Aldrich (USA). RPMI 1640 medium, DMEM medium, McCoy’s 5 A medium, fetal bovine serum (FBS), penicillin-streptomycin were purchased from Gibco (USA). All the other chemicals were purchased from commercial sources with analytical grade.

### Cell lines and cell culture

Human colon cancer cell line HCT116 and HT-29, human breast cancer cell lines MDA-MB-231 and MDA-MB-435s, human non-small cell lung cancer cell line H1299, human ovarian cancer cell line ES-2 were obtained from Shanghai Institute of Materia Medica, Chinese Academy of Sciences (Shanghai, China). MDA-MB-231 and MDA-MB-435s cells were maintained in DMEM medium. HCT-116 and HT-29 cells were maintained in McCoy’s 5 A medium. ES-2 and H1299 cells were maintained in RPMI 1640 medium. All culture medium were supplemented with 10% FBS and 1% penicillin/streptomycin. Cells were cultured in a humidified incubator under 5% CO_2_ at 37 ℃.

### Cell viability assay

The cells suspended in 100 µL/well medium were plated in 96-well plates at the density of 5400 cells per well and incubated for 24 h. The cells were respectively treated with different concentrations of NCS or CPT for 48 h. 20 µL of MTT (5 mg/mL) was added to each well and the cells were further incubated at 37 ℃ for 4 h. The supernatant was discarded and 100 µL of DMSO was added into each well to dissolve the formazan. The absorbance at 570 and 630 nm were measured with a microplate reader (Tecan Infinite M1000 PRO).

### Clone formation assay

2 ml of cell suspensions (250 cells/ml) were seeded into each well of 6-well plate and allowed to adhere for 24 h. Cancer cells were exposed to different concentrations of NCS and CPT for 10 days. Then the cells were washed with PBS for twice, fixed with methanol for 30 min, and stained with 0.1% crystal violet. The cells were observed and the images were acquired under the microscope (Olympus).

### Cell cycle analysis

Cell cycle analysis was analyzed using the cell cycle detection kit (Key GEN Bio TECH, China) according to the manufacturer’s protocol. 2 ml of cell suspensions (2 × 10^5^ cells/ml) were seeded in 6-well plates and allowed to adhere for 24 h. After the treatment with NCS and CPT respectively for 24 h, the cells were harvested and washed twice with cold PBS. Then the cells were fixed in 70% ice-cold ethanol and stored overnight at − 20 °C. The fixed cells were resuspended in binding buffer containing RNase and stained with PI in PBS in the dark for 30 min at room temperature. The stained cells were then analyzed using flow cytometry on an Accuri C6 flow cytometer (BD Biosciences, Franklin Lakes, NJ, USA). The distribution of cells in each phase of the cell cycle was determined by using FlowJo7.6.1 analysis software.

### Apoptosis analysis

Cell apoptosis was analyzed using the Annexin V-FITC Apoptosis Detection Kit (Key GEN Bio TECH, China) according to the manufacturer’s protocol. Briefly, 2 × 10^5^ cells were seeded in 6-well plates and allowed to adhere for 24 h. After the treatment with indicated concentrations of NCS and CPT for 48 h, the cells were collected and washed twice with cold PBS, then resuspended in a binding buffer containing Annexin V-FITC and PI. The cells were stained in the dark for 30 min at room temperature, and the fluorescence intensity was measured by using flow cytometry on an Accuri C6 flow cytometer.

### Western blotting analysis

The cells were seeded in 6-well plates at a density of 4 × 10^5^ cells/well. The next day, the cells were treated with indicated concentrations of NCS for indicated time periods. Following the treatment, the cells were harvested and lysed. Lysates were centrifuged and the protein extracts were quantified by BCA protein quantification kit (LIFE iLAB BIO, China). Equivalent amount of protein was subjected to sodium dodecyl sulfate-polyacrylamide gel electrophoresis and then transferred to PVDF membranes (Millipore) for immunoblotting. Everyblot blocking buffer (Bio-rad, USA) was used for blocking the membrane for 15 min at room temperature. The membranes were washed with TBS-T and then incubated with primary antibodies overnight at 4 °C. After washing with TBS-T buffer for 40 min, the membranes were incubated with horseradish peroxidase-conjugated secondary antibody (Santa Cruz, USA) for 2 h at room temperature. The proteins of interest were incubated with ECL substrate and detected by BIO-RAD ChemiDoc (Bio-rad, USA).

### Comet assay

HCT-116 cells (2 × 10^5^ cells/well) were seeded in 6-well plates. After the treatment with indicated concentrations of NCS and CPT, the cells were harvested, washed and centrifuged. Then the supernatants were removed and the cells were resuspended in PBS. Cells (1 × 10^6^ cells/mL) were combined with molten low point 2% agarose gel at 37 °C at a volume ratio of 1: 1. Then 50µL of this mixture was quickly pipetted onto the slides which pre-covered with 1% agarose gel. The slides were placed at 4 °C in the dark for 30 min, and then immersed in lysis solution (NaCl 2.5 M, Na_2_EDTA 100 mM, Tris 10 mM, 1% Sarkosyl, 1% Triton X-100, 10% DMSO, pH = 10) for 30 min at 4 °C in the dark. Next, the slides were placed in electrophoresis buffer to unwind for 30 min, and then subjected to electrophoresis at 25 volts for 30 min (Electrophoresis buffer: Na_2_EDTA 1 mM, NaOH 300 mM, pH = 13). Finally, the slides were stained with DAPI and visualized with a fluorescence microscopy (Olympus).

### Extracellular DNA cleavage assay

To make the 10 µl reaction systems, 7µL H_2_O, 1µL pHOT DNA, 1µL different concentrations of NCS or CPT, and 1µL reaction buffer was mixed and incubated at 37 °C for 30 min. Then 2µL stop buffer was added to stop the reaction. After that, all the samples were respectively added onto 1% agarose gel and electrophoresed with 1×TAE electrophoresis solution at 80 V for 20 min. The gel was stained with EB for 15 min, washed with double distilled water for 10 min, and then visualized under UV illuminator (BIO-RAD ChemiDoc, Bio-rad, USA).

### EB/Hoechst 33258-DNA fluorescence competition assays

For EB fluorescence competition experiment [31], certain amount of double-distilled water and EB were added to the cuvette, and then a sufficient and certain amount of ctDNA (calf thymus DNA) (ctDNA/EB = 4) was subsequently added to the reaction system. Different concentrations of NCS (0–50 µM) were respectively added into the cuvette and the emission spectrum change was detected by the fluorescence detector (RF-6000, Shimadzu, Japan). The protocol of Hoechst 33258-DNA (ctDNA/Hoechst 33258 = 32) fluorescence competition assay was the same as described above.

### Topo I mediated DNA relaxation assay and topo II mediated kDNA decatenation assay

The effects of NCS on topo I and topo II activities were examined by topo I mediated DNA relaxation assay and topo II mediated kDNA decatenation assay using topo I and topo II assay kits (TopoGEN, USA) according to the manufacturer’s protocol. Briefly, the topo I activity was tested in a 20 µL reaction system including 1 µL human Topo I, 2 µL 10× TGS, 1µL volume of NCS or CPT, 1 µL pHOT DNA, and variable volume H_2_O. The mixtures were incubated at 37 ℃ for 30 min. The reaction was stopped by adding 5 µL 5×stop buffer immediately. The samples were subjected to electrophoresis on a 1% agarose gel at 80 V. Then the gel was stained by EB and distained in water before being visualized under UV illuminator. CPT, a known topo I inhibitor, was served as the positive control [32, 33]. The effect of NCS on topo II activity was determined by using a similar procedure of topo I [34].

### Trapped in agarose immunostaining (TARDIS) assay

To quantify topoisomerase DNA complexes and determine whether NCS is a topoisomerase repressor, TARDIS assay [35, 38] was performed. Briefly, 2 × 10^5^ cells were seeded in 6-well plates and allowed for incubation at 37 °C overnight. After treatment with NCS for 2 h, the cells were harvested and washed with cold PBS for twice. Then the supernatant was removed and PBS was added to resuspend the cells. 50 µL of cell suspension (4 × 10^6^ cells/mL) was then mixed with an equal volume of 2% low melting point agarose gel at 37 °C, and the samples were spread evenly on a glass slide which was pre-covered with 1% agarose gel. All the slides were placed at 4 °C until fully solidified and then immersed in lysis buffer (1% SDS, 20 mM sodium phosphate, 10 mM EDTA, pH 6.5. Typically prepare 1 L) immediately for 2 h at 4 °C. The slides were placed in 1 M NaCl (containing protease inhibitor) solution at room temperature for 30 min to wash away the unbound protein. After washed with PBS (containing protease inhibitor) for three times, the slides were incubated with primary antibody overnight at 4 °C. After washing with PBST, the slides were incubated with the fluorescent-conjugated secondary antibody for 2 h at room temperature in the dark, and then washed with PBST. Finally, the slides were mounted with fluorescent mounting medium containing DAPI, and observed under a fluorescence microscope.

### Molecular docking study

The 3D structure of ligand NCS was modelled and energy-minimized by Chem3D. The 3D structure of topo I (PDB ID: 1EJ9) was fetch from PDB database. A blind docking was performed by Qvina-W and the interactions between ligand and receptor was analyzed and visualized using pymol [36].

### Statistical analysis

Data were presented as mean ± standard deviation (SD) of three separate experiments and analyzed via Student’s *t*-test. A p value less than 0.05 was considered as statistical significance.

## Conclusions

NCS, a member of amaryllidaceae alkaloids, possesses a potent antitumor effect. Previous studies showed that NCS could interact with the ribosomal subunit and inhibited the formation of peptide bonds [23], and NCS could induce the impairment of actin cytoskeleton organization by the GTPase RhoA and the elongation factor eEF1A [22]. In addition, a most recent study reported that NCS suppressed oral cancer metastasis by modulating cathepsin B and extracellular signal-related kinase pathways [39]. In this study, we found that NCS inhibited the ability of topo I to unwind DNA substrate, and it might be a topo I suppressor rather than a topo I poison. NCS tends to bind with topo I in a pocket composed of residues TYR211, ARG434, GLU208, ASP344, ASN345 and HIS346. The newly discovered mechanism of NCS has shown promising results in suppression and death of cancer cells. It is worth noting that, this new antitumor mechanism presents a more targeted approach and better understanding of the molecular pathways involved, and lays a good foundation for the development of anti-cancer drugs based on topo I inhibition. The discovery has great potential to lead to the development of new and effective cancer treatments in the future.

## Data Availability

The data supporting the findings of this study are available upon reasonable request from the corresponding author.
